# Understanding human gut diseases at single-cell resolution

**DOI:** 10.1093/hmg/ddaa130

**Published:** 2020-06-26

**Authors:** Emilia Bigaeva, Werna T C Uniken Venema, Rinse K Weersma, Eleonora A M Festen

**Affiliations:** Department of Gastroenterology and Hepatology, University of Groningen, University Medical Center Groningen, Hanzeplein 1, 9713GZ Groningen, The Netherlands

## Abstract

Our understanding of gut functioning and pathophysiology has grown considerably in the past decades, and advancing technologies enable us to deepen this understanding. Single-cell RNA sequencing (scRNA-seq) has opened a new realm of cellular diversity and transcriptional variation in the human gut at a high, single-cell resolution. ScRNA-seq has pushed the science of the digestive system forward by characterizing the function of distinct cell types within complex intestinal cellular environments, by illuminating the heterogeneity within specific cell populations and by identifying novel cell types in the human gut that could contribute to a variety of intestinal diseases. In this review, we highlight recent discoveries made with scRNA-seq that significantly advance our understanding of the human gut both in health and across the spectrum of gut diseases, including inflammatory bowel disease, colorectal carcinoma and celiac disease.

## Introduction

Since its early scientific investigations in the 1960s, we have accumulated a vast amount of knowledge on gut physiology and gastrointestinal diseases (1). The multifunctional nature of the human intestine, illustrated by its key role in food digestion, nutrient absorption and transportation, in immune response to pathogens and in forming a physical defense barrier, implies an exceptional biological complexity. Although tremendous scientific effort has been applied to grasp this complexity, it was not until recent technological advances like single-cell RNA sequencing (scRNA-seq) analysis that the cellular landscape of the human gut could be assessed at a high resolution. Single-cell transcriptomics has unveiled remarkable heterogeneity within major cell types and has identified new cell subpopulations that contribute to the complex intestinal cellular composition. Moreover, scRNA-seq has offered an unprecedented view of human disease by deconvoluting cellular interactions and pathway crosstalk that underlie disease pathophysiology (2). In this review, we discuss the findings of signature studies that employed scRNA-seq to profile cell types in normal gut mucosa (3) and in mucosa of patients with celiac disease (CeD) (4), inflammatory bowel disease (IBD) (5), including both Crohn’s disease (CD) (6,7) and ulcerative colitis (UC) (8–10), and colorectal carcinoma (CRC) (11–13), as detailed in Table 1. Since corresponding human data is as of yet unavailable, we also discuss a study of the mouse small intestinal epithelium that identified the cellular response to bacterial and helminth infections (14).

**Table 1:** Single-cell transcriptomic studies in human gut

Reference	Sample	Target cell population	Number of generated cells, sample size and identified cell types
*Healthy gut*			
Wang *et al.* 2019 (3)	Healthy donors,	Epithelial cells	• 14.537 cells—6 donors
	Mucosal biopsies of ileum, colon and rectum		• 7 epithelial cell subsets
*Celiac disease (CeD)*			
Atlasy *et al.* 2019 (4) *preprint*	Healthy controls and CeD	Immune cells	• 3.994 cells—6 donors and 8 CeD patients
	Mucosal biopsies of duodenum		• 5 main immune cell lineages (7 subsets of T cells, 1 subset of B cells, 2 subsets of plasma cells, 7 subsets of myeloid cells, 4 subsets of mast cells)
*Inflammatory bowel disease (IBD)*			
Huang *et al.* 2019 (5)	Healthy donors and IBD (UC, CD, IBD-U), all pediatric	Epithelial, stromal and immune cells	• 73.165 cells—pediatric: 6 donors, 6 IBD-U (colitis), 2 UC, 3 CD patients • 9 major cell types: epithelial cells (10 clusters), stromal cells (8 clusters), and 7 immune cell lineages (10 myeloid cell clusters, 7 B cell subsets, 2 plasma cell subsets, and 16 subsets of T and NK cells)
	Mucosal biopsies of colon		
Martin *et al.* 2019 (6)	CD	Stromal and immune cells	• 82.417 cells—11 CD patients
	Mucosal biopsies of ileum (matched inflamed and non-inflamed); peripheral blood		• 47 (33 if combining shared annotations) cell subsets: 8 stromal cell subsets, 25 immune cell subsets (from 7 distinct lineages)
Uniken Venema *et al.* 2019 (7)	CD	Immune cells	• 5.292 T cells—3 CD patients
	Mucosal biopsies of inflamed ileum and peripheral blood		• 6 distinct T cell subsets
Parikh *et al.* 2019 (8)	Healthy donors and UC	Epithelial cells	• 11.175 cells—3 donors, 3 UC patients
	Mucosal biopsies of colon (matched inflamed and non-inflamed UC mucosa)		• 10 epithelial cell subsets in healthy colon, 12 cell subsets in inflamed UC colon
Kinchen *et al.* 2018 (9)	Healthy donors and UC	Stromal cells	• 9.591 cells from 5 donors, 5 UC patients
	Mucosal biopsies of colon (matched inflamed and non-inflamed UC mucosa)		• 11 stromal cell subsets in healthy colon, 12 subsets in UC colon
	colonic organoids		
Smillie *et al.* 2019 (10)	Healthy donors and UC	Epithelial, stromal and immune cells	• 360.650 cells—12 donors, 18 UC patients
	Mucosal biopsies of colon (matched inflamed and non-inflamed UC mucosa)		• 51 cell subsets: 15 epithelial cell subsets; 13 stromal cell subsets, 23 immune cell subsets
*Colorectal carcinoma (CRC)*			
Li *et al.* 2017 (11)	CRC	Epithelial, stromal and immune cells	• 969 cells—resected primary tumors of CRC patients and 622 cells—the nearby normal mucosa of 7 of these patients
	Tumor tissue and matched adjacent normal mucosa of colon, rectum or caecum		• 7 distinct cells types: epithelial cells (9 clusters), stromal cells (3 subsets of fibroblasts, endothelial cells), immune cells (T cells, B cells, mast cells and myeloid cells)
Uhlitz *et al*. 2020 (12) *preprint*	CRC	Epithelial, stromal and immune cells	• ~50.000 cells—8 CRC patients
	Tumor tissue and matched adjacent normal mucosa of colon, rectum or caecum; matching CRC organoids		• 7 main types of epithelial cells, 5 tumor-specific epithelial cell subsets; stromal cells (pericytes, glial cells, endothelial cells and 5 subsets of fibroblasts); immune cell lineages (26 subsets in total assigned over T cells, B cells, plasma cells, myeloid cells and mast cells)
Zhang *et al*. 2018 (13)	CRC	Immune cells	• 11.138 T cells—12 CRC patients
	Tumor tissue and matched adjacent normal mucosa of colon and rectum; peripheral blood		• 20 T cell subsets
*Infectious disease (in mouse intestine)*			
Haber *et al.* 2017 (14)	Healthy mice and mice infected with *Salmonella enterica* or *Heligmosomoides polygyrus*	Epithelial cells	• 53.193 cells—small intestine and organoids of 2–4 mice per group • 15 epithelial cell subsets
	Dissociated cells from small intestine and epithelial organoids		

UC, ulcerative colitis; CD, Crohn’s disease; IBD-U, inflammatory bowel disease unclassified

This review (1) describes the key scRNA-seq findings in the three main cellular compartments of the intestinal mucosa—epithelial, stromal and immune—and (2) highlights cellular remodeling and cell–cell interactions in gut disease.

## Epithelial cell compartment

The intestinal epithelium lines the luminal surface of the gut mucosa and carries out a diversity of vital functions: it maintains a physical barrier, shielding the interior intestinal milieu from luminal content and pathogens, executes absorptive and metabolic tasks, controls bacterial growth and actively contributes to immune responses (15). Conventionally, we recognize undifferentiated intestinal stem cells, positioned at the crypt base, which via transit-amplifying (TA) cells give rise to the specialized intestinal cell lineages. These include absorptive enterocytes/colonocytes, enteroendocrine cells, goblet cells, Paneth cells and, less known, tuft cell-expressing receptors to sense luminal pathogens (16,17) and microfold cells (M-cells) guiding transport of luminal antigens to the lamina propria (18). Structural deviations in the epithelial compartment can cause intestinal barrier dysfunction that marks many intestinal disorders, including infectious diseases, inflammatory bowel disease (IBD), celiac disease (CeD) and colorectal carcinoma (CRC) (19,20). The scRNA-seq studies (1) identified a novel (*BEST4* expressing) absorptive cell type regulating pH balance (5,8,10), (2) showed the existence of Paneth-like cells in the colon (3,8), (3) distinguished an inflammation-associated subset of goblet cells (8), (4) highlighted the role of M-cells in disease (10) and (5) reported specific responses of epithelial cells to intestinal infection (14).

### BEST4 expressing absorptive cells

This newly identified distinct subpopulation of intestinal absorptive cells highly expresses the calcium-sensitive chloride channel bestrophin-4 (*BEST4)* and the pH detecting proton channel otopetrin 2 (*OTOP2)* and is therefore predicted to transport salt, ions and metals (8,10). By maintaining luminal pH, *BEST4/OTOP2* cells are thought to support optimal microbial growth, marking a novel component in the host–microorganism interaction. Moreover, BEST4+ cells are a previously unknown source of the paracrine hormone uroguanylin, which regulates intestinal electrolyte homeostasis by binding to the guanylyl cyclase C (GC-C) receptor and, thereby, increases intracellular levels of cyclic guanosine monophosphate (cGMP) (21,22). Dysfunctional cGMP/GC-C signaling has been implicated in compromised epithelial barrier function, increased intestinal inflammation and tumor growth (23), accelerating the progression of gastrointestinal disorders such as IBD and colon carcinoma (24,25). Single-cell profiling showed that both IBD (8,10) and CRC (11,12) are marked by the loss of *BEST4/OTOP2* cells, supporting the role of cGMP/GC-C dysregulation in these gut diseases.

### Paneth-like cells in the colon

Paneth cells, found in the crypt base in the small intestine, form a secretory lineage that is crucial for epithelial barrier function and epithelial cell renewal (26,27). These cells secrete antimicrobial peptides and factors that support intestinal stem cells. In contrast to the small intestine, healthy colonic crypts do not harbor Paneth cells and, therefore, rely on other sources for these factors. Colonic Paneth-like cells (PLCs) have been identified in mice but remained obscure in humans (28,29). Following up on a scRNA-seq study that describes a population of PLCs in the human colon (30), Wang *et al.* indeed verified the existence of PLCs in adult colon and showed that these cells, much like ileal Paneth cells, express genes involved in bacterial defense and genes that encode factors to sustain intestinal stem cells (3). Moreover, another scRNA-seq study detected a subset of crypt-base goblet cells that highly express the antimicrobial peptide lysozyme (*LYZ*) in inflamed colon and which most likely act as PLCs (8). While impaired Paneth cell function has been shown to contribute to the pathogenesis of ileal CD and CeD (31–33), the involvement of colonic PLCs in gut diseases is yet to be elucidated.

### Inflammation-associated goblet cells

Luminal secretion of mucins by goblet cells is critical for the establishment of a chemical and physical barrier as a frontline of innate host defense (34). Dysregulated goblet cell function contributes to barrier breakdown in UC (35) and CeD (36); however the pathways that underlie this breakdown are still unknown. ScRNA-seq studies mapping the cells of colonic epithelia reveal an exceptional goblet cell diversity, distinguishing several subsets of varying maturity and localization within the intestinal crypts (8). There appears to be a positional remodeling of goblet cells in IBD, along with the emergence of a disease-associated subset of goblet cells in inflamed colon. Moreover, the goblet-cell-secreted antibacterial defense factor WFDC2 is lost in active UC, suggesting a novel functional role of this factor in the maintenance of the mucosal barrier.

### The role of M-cells in disease

M-cells contribute to the adaptive immunity in the gut by delivering luminal antigens to the underlying mucosal lymphoid tissues (37). While M-cells normally reside in the follicle-associated epithelia of the small intestine and are rarely found in healthy colon, scRNA-seq shows that M-cells markedly expand in the inflamed colon of UC patients (10). Activated M-cells highly express chemokines recruiting immune cells to the site of inflammation. These specialized epithelial cells highly express a large number of genes known to be associated with IBD susceptibility, pinpointing M-cells as a central node in the cell–cell interaction network during IBD inflammation (10). Besides inflammation, infectious conditions have been shown to ectopically induce M-cells, where they act as a portal for pathogen invasion in the mucosa (38). The only available scRNA-seq study that investigated responses of epithelial cells during intestinal infection was limited to mice and could not detect M-cells at the resolution of their data, and therefore, this study was unable to report infection-induced changes in M-cells (14).

**Table 2:** Fibroblast subtypes in the human gut identified by single-cell transcriptomics

Subset	Gene markers^*^	Location/function	Reference (subset annotation)^**^
1. Myofibroblasts	*MYH11*, *ACTG2*, *DES*	Distributed throughout the lamina propria Express contractile genes Relatively unchanged in inflammation	(5) Huang *et al.* (6) Martin *et al.* (smooth muscle cells) (9) Kinchen *et al.* (10) Smillie *et al.* (12) Uhlitz *et al.*
2. Lamina propria fibroblasts	*CCL2*, *CCL8*, *CCL11*, *CCL13*, *CXCL1*, *APOE*, *ADAMDEC1*	Distributed throughout the lamina propria; Involved in structural organization of extracellular matrix	(5) Huang *et al.* (6) Martin et al. (fibroblasts) (9) Kinchen *et al*. (S1) (10) Smillie *et al.* (WNT2B^+^ Fos^hi/lo^ fibroblasts) (12) Uhlitz *et al.* (fibroblasts)
3. SOX6^+^ fibroblasts	*SOX6*, *F3*, *WNT5A*, *WNT5B*, *BMP2*, *BMP4*, *FRZB*	Reside in a close proximity to epithelial cells (near the villus) Regulate epithelial regeneration	(5) Huang *et al.* (epithelial proximal fibroblasts) (9) Kinchen *et al*. (S2) (10) Smillie *et al.* (WNT5B^+^ fibroblasts) (12) Uhlitz *et al.* (upper crypt fibroblasts)
4. RSPO3^+^ fibroblasts	*RSPO3*, *S3*, *S7*, *WNT2B*, *TNFRSF13B*	Decrease upon inflammation Reside near the crypt Regulate the survival of intestinal stem cells	(5) Huang *et al.* (WNT2B^hi^ and TNFRSF13B^+^ fibroblasts) (9) Kinchen *et al.* (S3) (10) Smillie *et al.* (12) Uhlitz *et al.* (crypt-base fibroblasts)
5a. Inflammation-associated fibroblasts (IAFs)	*IL6*, *IL11*, *CXCL3*, *CXCL5*, *CXCL6*, *MMP3*, *MMP10*, *CHI3L1*, *OSMR*	Almost exclusive for inflamed mucosa (in IBD) Mobilize the immune response	(5) Huang *et al.* (inflammatory fibroblasts) (6) Martin *et al.* (activated fibroblasts) (9) Kinchen *et al.* (S4)^#^ (10) Smillie *et al.*
5b. Cancer-associated fibroblasts (CAFs)	*TGFB1*, *TGFB3*, *MMP2*, *MMP3*, *MMP11*	Exclusive for tumor tissue (in CRC) Produce multiple pro-oncogenic growth factors Mediate paracrine responses in tumors	(11) Li *et al.* (12) Uhlitz *et al*.

^*^selected subset-defining gene markers that overlap in the studies listed under ‘Reference’.

^**^indicated only if subset annotation differs from the one indicated under ‘Subset’.

^#^IAFs described by Kinchen *et al.* had a mixed gene expression signature of RSPO3^+^ fibroblasts and IAFs when compared to the clusters described by Smillie *et al.* and Huang *et al.*

5a and 5b divide a category of disease-associated subsets of fibroblasts: IAFs in IBD and CAFs in CRC.

GO, gene ontology; IBD, inflammatory bowel disease; CRC, colorectal cancer.

### Epithelial response to the intestinal infection

ScRNA-seq reveals that the restructuring of the epithelial barrier, involving shifts in cell proportions and cell-intrinsic programs, is specific to the identity of the pathogen (14). For instance, goblet and tuft cells—secretory cells that are known to respond to parasites—accumulate in mouse small intestine during helminth infection, whereas the proportions of absorptive enterocytes and Paneth cells increase in response to Salmonella infection. The question whether these findings translate to the human gut warrants further investigation.

Lastly, single-cell profiling of tumors and matched normal tissues provides a unique opportunity to identify changes in the epithelial cell compartment in CRC. Two scRNA-seq studies describe a pronounced expansion of undifferentiated stem-/TA-like cells within tumors, comprising more than 90% of all tumor epithelial cells (11,12). While stem cells are essential for tissue homeostasis and regeneration, they also drive therapy resistance in cancer. Tumor-specific stem-/TA-like cells show higher expression of bottom-crypt markers than cells in the normal colon epithelium, have high proliferative activity and express genes linked to oncogenic processes (11,12). These scRNA-seq findings imply that epithelial cells in CRC display considerable cell plasticity and have multilineage differentiation capacity.

## Stromal cell compartment

Residing within the intestinal lamina propria, stromal cells such as fibroblasts, myofibroblasts, pericytes and endothelial cells provide a supportive matrix for the epithelium. Stromal cells dynamically interact with both epithelial and immune cells, playing crucial roles in regulating epithelial barrier homeostasis, gut innate immunity, tissue repair and tumor development (39,40). Recently, scRNA-seq studies profiling gut mucosal cells revealed previously unknown heterogeneity within the stromal compartment. In addition, these studies identified new and distinct intestine-specific mesenchymal subsets and uncovered their functional role to maintain and regenerate the intestinal epithelium in health and disease.

Among stromal transcriptomes, most studies distinguish the following distinct fibroblast subsets along the crypt–villus axis of the human gut: myofibroblasts, lamina propria fibroblasts, SOX6^+^ (upper crypt) fibroblasts, RSPO3^+^ (crypt base) fibroblasts and disease-associated subsets of fibroblasts (Table 2). These fibroblast subtypes show transcriptional, spatial and functional diversity. Lamina propria fibroblasts were shown to diffusely populate the mucosal connective tissue and express non-fibrillar collagens and elastic fibers. In turn, fibroblasts that characteristically express transcription factor *SOX6* and Wnt ligands *WNT5A* and *WNT5B* reside in close proximity to the epithelial monolayer, suggesting their role in epithelial cell proliferation and differentiation and, hence, in epithelial barrier maintenance. Another fibroblast subset is defined by the expression of *RSPO3*, *WNT2B* and *TNFRSF13B* and spatial proximity to the crypt base, regulating the survival of intestinal stem cells. Single-cell studies show that the abovementioned fibroblasts can be detected in normal gut mucosa as well as in inflamed mucosa of IBD patients (5,9,10) and in tumors of CRC patients (12). Two specific disease-associated fibroblast types have been identified: inflammation-associated fibroblasts (IAFs) in IBD, which are almost exclusively present in inflamed mucosa and appear to play an important role in recruiting immune cells to the gut mucosa, and cancer-associated fibroblasts (CAFs) that generally seem to play a tumor-promoting role producing pro-oncogenic growth factors.

## Immune cell compartment

The gut is the largest immune organ in the human body, and the mucosal immune system is crucial in health and disease, as it guards the barrier between the body’s internal milieu and the microbiome in the gut lumen (41). Although many mucosal immune cells are gut-resident and their main role is maintaining homeostasis, scRNA-seq studies provide additional evidence for their active involvement in inflammation and carcinogenesis.

ScRNA-seq highlights T cells as the most functionally diverse and flexible immune cells in the human gut. Instead of the classic denomination based on surface markers (i.e. CD4-CD8), scRNA-seq differentiates cells based on their gene expression, classifying T cells based on their origin, spatial localization and function. Under homeostatic circumstances, the gut mucosa harbors a vast reservoir of naive, central memory and resident memory T cells. In disease, the number of specific T cell subsets expands, bearing out the fluidity and the functional diversity of the compartment (6,10). Studies that employ scRNA-seq to profile human gut cells in CRC show similar inflammatory responses as have been observed in IBD (11–13). In active IBD, tissue-resident T cells fulfill a multitude of different functions: pro-inflammatory—through cytotoxic (*TNF*, *IFNG*) or antimicrobial (*IL22*, *IL17A*) pathways, and anti-inflammatory—through suppressive pathways (*IL10*, *TIGIT*). Still, separate populations of cytotoxic T cells and regulatory T cells (Treg) are clearly present in IBD. While classically cytotoxic T cells are CD8^+^ T cells, scRNA-seq reveals that on gene expression level, cytotoxic T cell subset consists of both CD4^+^ and CD8^+^ T cells (7,10). Regulatory T cells, as characterized by the expression of *IL10* and *CTLA4*, are present in the healthy gut and expand during inflammation.

Furthermore, scRNA-seq has provided new insight into *IL17* expressing cells, which are known to play a central role in chronic inflammation in IBD (42). Although these cells are classically identified as one Th17 cell population, scRNA-seq provided evidence for the existence of a much wider array of Th17 cell subtypes (6,7,10). Thus, Th17 cell subtypes are ranging from classic Th17 CD4^+^ T cells, which have an inflammation-modulating phenotype and appear to share a lineage with Treg cells, to the Th17-like cells, with a cytotoxic phenotype, on the other end of the spectrum. The latter are a mixed population of CD4^+^ and CD8^+^ T cells. ScRNA-seq detected the marked expansion of this population in the gut mucosa in both IBD and CRC, and it seems to play an important role in aggravating tissue damage and subsequent cancer progression (10,12,13).

Along with the T cells, B cells are a very abundant immune population in the gut mucosa, which further increases in numbers upon active inflammation. Moreover, in active IBD many B cells evolve into plasma cells, favoring IgG producing phenotype over IgA (5,10), which is consistent with the immunoglobulin class switching known in IBD.

ScRNA-seq characterized myeloid cell populations and demonstrated that myeloid cells exist on a scale of active development from monocytes to dendritic cells (DCs) and macrophages (Mfs). DCs survey the mucosa by sampling antigen, and scRNA-seq shows that monocyte-derived DCs form a stable population in the human intestine under homeostasis (43). Once activated, DCs migrate to the lymph nodes to interact with T and B cells. ScRNA-seq reveals that activated DCs, as characterized by the expression of NFκB-inducing cytokines and lymph-attracting chemokines, are more numerous in the mucosa of patients with IBD than in healthy controls (6). Likewise, gut-resident Mfs represent the most abundant mononuclear phagocytes in the body under physiological conditions, and activated pro-inflammatory Mfs are overrepresented in the gut mucosa of IBD patients (6,10,43). These activated DCs and pro-inflammatory Mfs have a central role in IBD, perpetuating disease activity independently of the adaptive immune inflammatory mechanisms targeted by anti-TNFα therapy (6).

## Functional networks

In healthy gut mucosa, cells from the different compartments interact to maintain gut barrier function. For instance, together Paneth(like) cells, BEST4^+^ cells and lamina propria (myo)fibroblasts stimulate epithelial cell renewal, while gut-resident DCs surveil the epithelium for invading antigens, and Mfs and T cells maintain the immune barrier. In disease, this well-orchestrated functional homeostasis is disturbed and remodeled.

**Figure 1:**
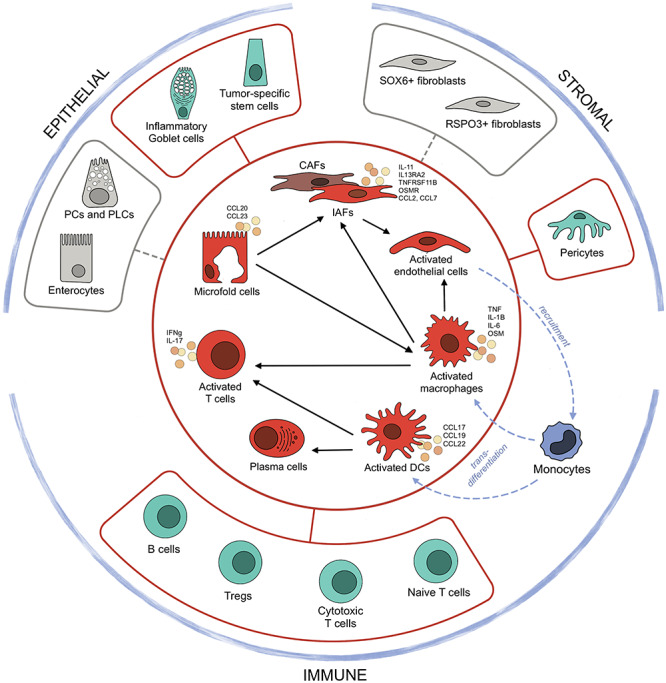
Gut disease-specific features identified by single-cell transcriptomics. The inner circle includes the cell types (in red color) that expand in disease and form key network hubs, mediating inter-lineage crosstalk. The outer circle highlights changes in the proportions of cell subtypes in each compartment that don’t directly contribute to the disease-associated cell–cell network but yet have detrimental effects for intestinal barrier homeostasis. Cell types in gray are depleted in active disease, while cell types in green considerably expand. Cell–cell interactions and their direction are marked by black arrows. Blue dashed arrows delineate the recruitment of circulating classical monocytes by activated endothelial cells, which in turn differentiate into pathogenic activated macrophages and DCs. Annotations: IAFs, inflammation-associated fibroblasts; CAFs, cancer-associated fibroblasts; DCs, dendritic cells; PCs, Paneth cells; PLCs, Paneth-like cells.

One of the strengths of scRNA-seq is that it enables the construction of functional cellular networks in health and in disease while pinpointing the central network hubs. Figure 1 outlines major disease-associated changes in cell composition of the intestinal mucosa and maps cell–cell interaction formed in human gut disease. ScRNA-seq nominates disease-associated cell subsets, such as IAFs/CAFs, M-cells, activated endothelial cells, activated Mfs, activated DCs and inflammatory T cells, as central hubs in the cross-lineage network that drive epithelial barrier breakdown and aggravate disease progression.

Furthermore, scRNA-seq defines the cellular remodeling in the three main intestinal cell compartments (epithelial, stromal and immune) during disease. Enterocytes and Paneth(like) cells in the epithelium, and SOX6^+^ and RSPO3^+^ subsets of fibroblasts in the stroma, whose functioning is essential for intestinal homeostasis, have been found to be depleted in inflamed mucosa, reflecting reduced compartmentalization in the diseased gut. On the other hand, scRNA-seq detected the expansion of pericytes, inflammatory goblet cells in IBD and tumor-specific stem cells in CRC. Even more pronounced changes have been described for the immune compartment, where naive T cells, cytotoxic T cells, Tregs and B cells largely contribute to the increased pool of immune cells at the site of inflammation.

## Discussion

Single-cell studies have shown that there is a remarkable cellular diversity between patients with similar phenotypes: single-cell transcriptome signatures stratify CRC tumors into subgroups with distinct patient survival (11) and stratify CD patients with ileal inflammation into subgroups with distinct response to anti-TNFa therapy (6). Molecular phenotyping will thus become a crucial step in personalized medicine, and further exploration of pathophysiological diversity in diseases of the gut will greatly improve our ability to realize this personalized medicine. At the same time, single-cell techniques are evolving further, first of all, allowing for higher throughput and lower cost per sample (44). Other new developments in high-resolution transcriptome-wide technologies are capable to infer the spatial localization of the cells of which gene expression is measured, shedding more light on the functioning of the gut mucosa as an organ (45,46). Single-cell technologies revolutionized the way we approach human biology, culminating in an exciting effort to map all human cells as championed by the Human Cell Atlas (https://www.humancellatlas.org). Consequently, defining human gut at single-cell resolution will continue to reshape our understanding of gastrointestinal health and disease.

*Conflict of Interest statement.* None declared.

## Funding

R.K. Weersma is supported by a Diagnostics Grant from the Dutch Digestive Foundation (D16–14). E.A.M. Festen is supported by a MLDS Career Development grant (CDG 14–04).
